# Intravascular Imaging in Ultra-Low or Zero-Contrast Percutaneous Coronary Interventions: The Time Is Now?

**DOI:** 10.3390/jcm12237499

**Published:** 2023-12-04

**Authors:** Kyriakos Dimitriadis, Nikolaos Pyrpyris, Aggelos Papanikolaou, Eirini Beneki, Panagiotis Tsioufis, Alexios Antonopoulos, Christos Fragoulis, Fotis Tatakis, Georgios Koutsopoulos, Konstantinos Aznaouridis, Konstantina Aggeli, Konstantinos Tsioufis

**Affiliations:** First Department of Cardiology, School of Medicine, National and Kapodistrian University of Athens, Hippokration General Hospital, 115 27 Athens, Greece; npyrpyris@gmail.com (N.P.); agepap25@otenet.gr (A.P.); e.beneki@hotmail.com (E.B.); panos.tsioufis@gmail.com (P.T.); alexios.antonopoulos@cardiov.ox.ac.uk (A.A.); christosfragoulis@yahoo.com (C.F.); fotistatakis@yahoo.gr (F.T.); giorgoskoutsopoulos93@gmail.com (G.K.); conazna@yahoo.com (K.A.); dina.aggeli@gmail.com (K.A.); ktsioufis@gmail.com (K.T.)

**Keywords:** percutaneous coronary intervention, intravascular imaging, contrast media, intravascular ultrasound, contrast-induced nephropathy

## Abstract

Ultra-low contrast percutaneous coronary interventions (ULPCIs) are a novel field of interventional cardiology, aiming to reduce the risk of contrast-induced nephropathy (CIN), which is a well-described adverse event after angiography. CIN is a well-described adverse event following PCI, especially in high-risk patients, i.e., patients with an already deteriorating renal function or chronic kidney disease, as well as patients of advanced age or requiring an increased amount of contrast during their intervention. Among the techniques described for ULPCI procedures, intravascular imaging guidance seems a promising option, as it allows lesion recognition and characterization, stent implantation, and PCI optimization. Intravascular ultrasound (IVUS) is the modality most commonly used, as it does not require contrast injection, contrary to optical coherence tomography (OCT). Several clinical trials, assessing IVUS in the context of ULPCI, have shown that it can be safely used in this setting while offering a substantial reduction in contrast media volume, as well as renal adverse outcomes. This review aims to describe the need for ULPCI and technical considerations regarding the use of intravascular imaging in this setting, as well as analyze the available evidence from clinical trials regarding the safety and efficacy of IVUS-ULPCI, in order to provide a comprehensive summary for practicing physicians.

## 1. Introduction

Since the inception of percutaneous coronary interventions (PCI), contrast media has been a necessary means for rendering the coronary vessels under fluoroscopy. Contrast agents are iodine-based solutions used to visualize the coronary arteries during angiography, as they create a contrast between the vessels and their surroundings, thus providing better visualization and discrimination of the coronaries, which are necessary for lesion identification, assessment, and intervention [1]. Contrast media are well used for a handful of radiology studies of the human body; however, it is well known that these agents are associated with several adverse reactions, ranging from allergy to nephrotoxicity and contrast-induced nephropathy (CIN) [2]. Especially as, in the modern era of PCI, patients undergoing such procedures are older and have more comorbidities, there is a need not only to limit contrast volume to the safest volume achievable but also to identify those who are at increased risk of adverse events and would benefit from an ultra-low or even zero-contract PCI. Furthermore, complex interventions, in regards to difficult and multiple-lesion revascularization, and chronic total occlusion (CTO) revascularization require an increased amount of contrast injection, in order to ensure the best angiographic outcome [3]. These considerations led to efforts to reduce the use of contrast in specific patients’ categories in order to diminish adverse outcomes and provide an alternative to patients requiring more complex interventions, but patients are at increased or prohibitable risk for nephrotoxic adverse events, following the great volume of contrast that such interventions require.

Ultra-low contrast PCI (ULPCI) has recently emerged as a safe alternative for patients who cannot undergo a coronary intervention with the standard use of contrast media. Therefore, ULPCI is defined as an intervention, where the ratio of contrast media injected to the estimated glomerular function rate (eGFR) is lower than 1 (contrast volume/eGFR < 1) [4]. However, in general, ULPCI can be considered any contrast-limiting strategy, with the use of other methods in order to better visualize coronary anatomy, reaching up to zero-contrast PCI strategies in extremely high-risk patient phenotypes. Currently, both American and European guidelines on myocardial revascularization recommend the limitation of contrast use in patients with CKD or at high risk for CIN, as measured by adequate risk scores; however, the only measures supported are pre-procedural hydration and statin administration and the use of as low as possible low- or iso-osmolar contrast media, as other ULPCI techniques described in literature do not have enough evidence to be supported in guidelines [5,6,7]. These techniques include a number of interventional strategies for limiting contrast use, aiming to reduce operator dependence on contrast in order to visualize the coronary arteries. Among those, dual-plane computed tomography (CT) scanners and dynamic coronary roadmap (DCR) can significantly reduce contrast media volume compared to plain angiography and physiology guidance [8,9]. DCR offers automatic generation of a real-time overlay of the coronary arteries on fluoroscopy, based on a baseline angiography study. This tool can be used alongside other imaging techniques in order to advance guidewires and precisely position balloons and/or stents in the lesion, without the need for contrast injection. DCR has been associated in two available studies [8,9] with both high technical success and safety, as well as limited contrast use, thus it can evidently assist the operator in intervening under ULPCI conditions. Furthermore, co-registration of fluoroscopic and physiological parameters can help limit the use of contrast while creating a precise physiological-anatomical map of the coronaries [10]. With the emergence of intravascular imaging, the option of intravascular ultrasound (IVUS)-guided PCI, in the context of ULPCI, has emerged. IVUS is an imaging modality that can accurately depict the coronary arteries and create an “anatomical tree” of the coronaries, which can be used to interpret coronary lesions in the absence of contrast media. The independence of IVUS from contrast media injections creates a favorable advantage for the technique for use in ULPCI settings. Therefore, several clinical trials were designed in order to evaluate the technique in this setting and provide data regarding the safety and efficiency of an IVUS-guided procedure.

This review, thus, aims to provide a novel and comprehensive summary of the need for ULPCI and the advantages of an intravascular imaging-guided intervention while providing both technical guidance and consideration as well as an analysis of the available clinical data regarding the use of intravascular imaging in this field.

## 2. The Need for ULPCI—Patient Characteristics

**Figure 1:**
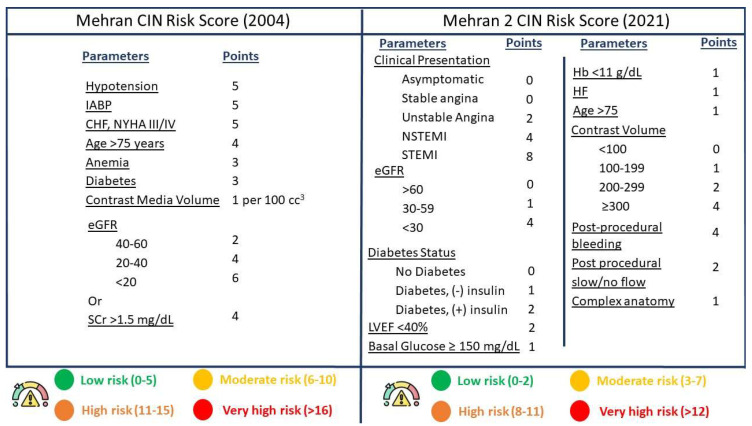
The Mehran 1 and 2 CIN risk scores, based on pre–procedural and procedural characteristics. These scores provide an estimation of the risk for adverse renal events after percutaneous coronary interventions. Abbreviations: CIN: contrast–induced nephropathy, IABP: intra–aortic balloon pump, HF: heart failure, CHF: congestive HF, NYHA: New York Heart Association, eGFR: estimated glomerular filtration rate, SCr: serum creatinine, STEMI: ST elevation myocardial infarction, NSTEMI: non–STEMI, LVEF: left ventricular ejection fraction, Hb: Hemoglobin.

ULPCI has emerged as a necessity for optimally managing patients at high risk for CIN. CIN can be defined as post-intervention renal impairment, as depicted by a 25% or 0.5 mg/dL increase of creatinine levels, compared to baseline, within 48–72 h after the administration of the contrast solution [11]. However, another definition for CIN has been introduced by a Kidney Disease Improving Global Outcomes (KDIGO) position paper, which defines “Contrast-Induced Acute Kidney Injury” as: (a) Plasma creatinine levels that have increased by 50% over the period of 7 days after the injection of contrast; (b) serum creatinine levels that are increased by 0.3 mg/dL, compared to baseline, in the first 48 h post-injection; or (c) urinary volume less than 0.5 mL/kg/h for over 6 h after the intervention [12]. The incidence of CIN varies among studies and definitions; however, a recent meta-analysis of clinical trials regarding the incidence of CIN post-angiography revealed an incidence of 9.6%, while there was a need for dialysis in 0.6% of patients [13]. Other clinical trials report a slightly increased incidence in patients undergoing PCI [14,15], which can possibly be attributed to the increased risk phenotype of patients undergoing coronary interventions in comparison to patients undergoing angiography for other indications. There are several risk factors predicting the increased risk of developing CIN after an intervention where contrast has been used. In particular, the most important risk factor is the presence of renal failure or chronic kidney disease (CKD) at baseline [14,16]. CKD is a common comorbidity among patients with coronary artery disease, with an estimated incidence of 25–46%, depending on the specific study characteristics and population [17,18,19]. Despite CKD patients being considered at high risk for undergoing PCI in general, recent data show that the number of coronary interventions in CKD patients has substantially increased over the years as more experience and data are gained in this patient category [20]. Therefore, this high-risk parameter for CIN is increasingly met in modern-era PCI patients, ultimately increasing their overall risk for adverse events, such as CIN. Other described risk factors include older age, heart failure, diabetes, anemia, atrial fibrillation, acute coronary syndrome presentation, and volume of contrast injected [15,21,22]. As there are a number of parameters contributing to an individual’s risk of developing CIN, several risk scores have been validated over the years in order to facilitate clinical decision making [23,24,25]. The most commonly used risk score in clinical practice is the Mehran CIN risk score. This score, created by Mehran et al. [26], uses a number of post-procedural parameters associated with the patient’s status, comorbidities, procedural contrast use, and renal function. The score discriminates each patient into four distinct risk categories, with those at high and very high risk having a CIN risk of 26.1% and 57.3%, respectively, while they are also associated with a risk for dialysis of 1.09% and 12.6%, respectively. Recently, the Mehran 2 score, which, in comparison to the original score, elaborates more on preprocedural and peri-procedural and has also been tested in patients with shock and myocardial infarction (Figure 1), showed a good discrimination of patients at high risk for CIN [23]. Finally, CIN is associated with worse patient outcomes, both regarding metabolic and renal physiology, where the need for dialysis may be necessary, as well as other procedural-related adverse events, including recurrence of myocardial infarction, bleeding events, and worse short- and long-term survival rates [27,28,29,30].

Despite CKD patients representing the largest category of patients, where ULPCI is most likely to be pursued and more commonly represented in ULPCI trials, there are also a number of other patient phenotypes that could benefit from such strategies. Patients with contrast media allergies are at increased risk for allergic reactions and anaphylaxis, which can result in increased mortality if left untreated. Even though pre-medication with corticosteroids has been described, in order to prevent such events [31], it is safer to consider an ultra-low or even zero-contrast PCI strategy in order to ensure no exposure to the allergen. Despite the lack of trials specifically assessing this patient scenario for ULPCI, a number of case reports [32,33,34,35] document the safety and feasibility of the technique without limiting the procedural success of the coronary intervention. Furthermore, in complex coronary interventions, especially in multimorbid patients, there could be a benefit to implementing ULPCI strategies. In particular, the independence of the operator from contrast injections could assist in completing the revascularization of all lesions in one intervention rather than being limited in stage-up procedures due to reaching the limits of contrast injection, especially in long procedures involving multiple lesions and CTOs. Finally, patients with sudden coronary artery dissection (SCAD) or iatrogenic dissection could benefit from a ULPCI, as contrast injections should be limited to the absolute necessary in order to not expand the dissected flap. Therefore, not only patients at high risk for CIN but also a large number of PCI patients could ultimately benefit from contrast-limiting strategies without reducing the success rate of the coronary intervention. In particular, a common, suitable approach for pursuing a ULPCI strategy patient phenotype would be a multimorbid, older patient with impaired renal function, CKD, or high risk of CIN and with multiple, complex lesions, which would require increased contrast use during the intervention in order to be revascularized in the index procedure and not be staged. Despite the complexity of these patients, available evidence shows the safety and efficacy of ULPCI in the setting of complex multivessel lesions, left main lesions, and CTOs [36,37,38,39]. However, the operator should be aware of the limitations of ULPCI methods, i.e., poor discrimination of calcified lesions with IVUS, and select the appropriate technique that would facilitate ULPCI.

## 3. Intravascular Imaging for ULPCI—Technical and Procedural Considerations

Intravascular imaging is a relatively novel aid to coronary interventions, which, by using different image acquisition technologies (i.e., ultrasound, optical coherence) intravascularly, assist operators in assessing intracoronary artery features, such as lesion length, severity, and characterization, and in optimizing PCI by precisely providing information on stent expansion and apposition. The use of such techniques in optimizing PCI outcomes is well described, while it is widely suggested to further implement intravascular imaging in routine practice in order to achieve the best patient and procedural outcomes [40]. In ULPCI settings, the most appropriate imaging modality is IVUS, which requires no contrast injections, as compared to optical coherence tomography (OCT), which requires a large amount of contrast for image acquisition [41]. IVUS is a sound-based (ultrasound) endovascular imaging modality that can acquire a live 360-degree cross-sectional image of the vascular system and thus the coronary arteries. Due to the different echogenicity of structures within the coronary artery wall, IVUS can display differences in the coronary artery wall and characterize coronary artery plaques. Furthermore, IVUS can assist in determining the most appropriate stent landing zone and visualize stent characteristics post-implantation, especially with regard to stent expansion. Image acquisition is performed via a manual or mechanical probe pullback in order to depict in a live and recorded echocardiographic image each coronary vessel and further assist in determining the length and severity of stenosis in the entirety of the coronary artery tree [40]. 

**Figure 2:**
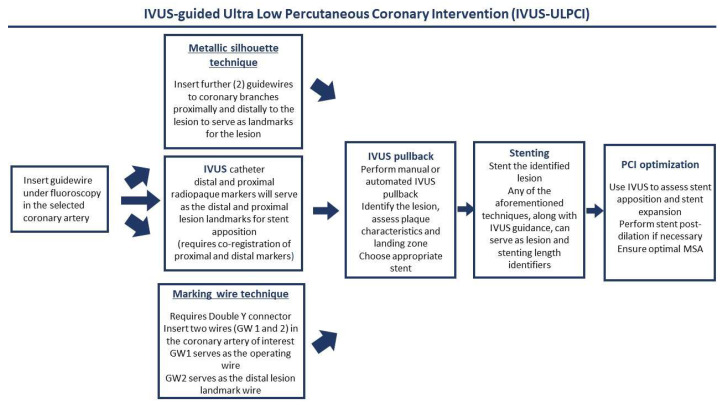
This algorithm provides an overview of selected techniques commonly used for ULPCI procedures. There are several techniques (i.e., marking wire, metallic silhouette) that aim to highlight the lesion’s borders, based on baseline angiography. Following IVUS pullbacks can be used to further identify the lesion and assess its characteristics. Following, stenting should be performed under IVUS guidance, while post-stenting PCI optimization, ensuring optimal stent expansion, should be used to enhance patient outcomes.

In the context of ULPCI, the basics of the use of IVUS remain unchanged. However, given the lack of contrast injection, in order to identify important anatomical landmarks as well as the optimal position of the probe, modifications to traditional practice algorithms need to be made. Several techniques have been methodologically described, mostly in clinical trials available in this specific setting [36,37,42] (Figure 2). To begin with, a guidewire needs to be advanced in the selected coronary artery, past the lesion of interest. Supplementary guidewires can be advanced to adjacent side branches in order to serve as landmarks and create a metallic silhouette. Another technique is to involve the “marking wire technique”, where using a Y connector, an operating and a marking guidewire are delivered, with the marking wire serving as the landmark for the distal lesion border, as recognized by IVUS, the operating guidewire serving for the stent delivery, and the IVUS probe as an identifier of the proximal lesion end. After identifying the coronary anatomy, with the use of the guidewires, IVUS pullbacks should be performed in order to identify potential lesions. After lesion identification using the aforementioned landmarks, stent delivery should be performed using the IVUS-acquired images. If no landmark guidewires were used to identify the lesion borders, the IVUS probe should be used to acquire two images (proximal and distal end), which should be projected at the time of stent apposition to help guide the operator between the lesion borders. Following stent expansion, post-expansion evaluation should also be made by IVUS in order to ensure optimal stent apposition. In general, all interventions are to be made under IVUS guidance, and contrast injection should be zero (zero-contrast PCI) or limited to the lowest amount possible (ULPCI). Regarding complications that cannot be assessed by IVUS, such as distal embolization, some trials mention the use of a final, low-dose, single-contrast injection in order to identify this complication [36,38]. However, others suggest that this should only be made when there is strong clinical evidence of distal embolization in order to follow an “absolute zero contrast” strategy [37]. Both strategies have been used in clinical trials; however, no trial to date compares a ULPCI to a zero-contrast strategy. In other words, it is still uncertain if a small contrast injection could have deleterious effects on these patients. It is evident that a final injection could provide safety to the operator and ensure optimal outcomes. As data on the field are still emerging, operators should always use a final injection with respect to their judgement, clinical evidence suggestive of adverse events, and their certainty of an optimal procedural outcome. Finally, it should be recognized that there are some limitations to the sole use of IVUS in such patients. Specific coronary anatomies, such as dilated vessels, heavily calcified lesions that result in IVUS signal dropout, and extreme vessel tortuosity and angulation, which could harden the insertion of the IVUS probe, may not result in optimal procedural outcomes; therefore, other techniques for ULPCI should be implemented. 

**Figure 3:**
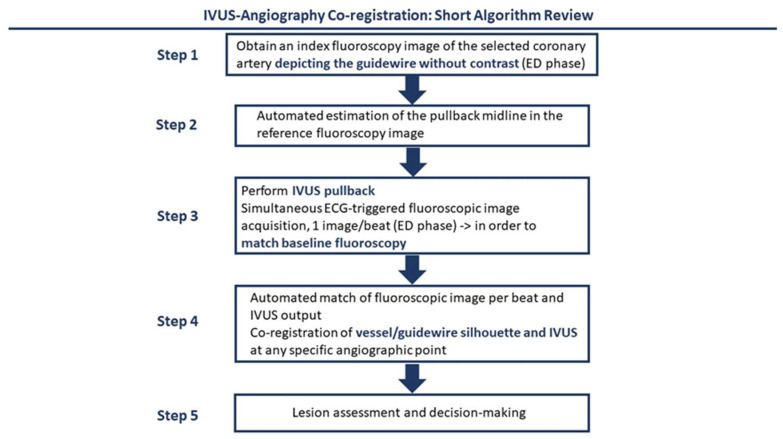
This algorithm provides an overview of co-registration of angiography and IVUS images. Baseline angiography images, at the end-diastolic (ED) phase, are used. Then, an automated estimation of the pullback midline is done, which assists the operator with software to match the IVUS pullback with angiography data. After IVUS pullback, software-created co-registered images are created, which can be used by the operators for lesion assessment and revascularization.

Recently, a new technology aims to combine angiographic images with IVUS. Co-registration is a novel technique where the fluoroscopic and IVUS images are automatically co-registered, allowing for simultaneous evaluation of the cross-sectional IVUS images along the coronary artery of interest, which is depicted in the angiogram [43] (Figure 3). In terms of technique, in non-ULPCI settings, a reference frame from an index angiogram, where contrast has been used, is selected, ideally at the end-diastolic phase of the cardiac cycle. Then, automated software identifies the ideal pullback line in the reference vessel. Following, IVUS pullback is performed, while simultaneously ECG-triggered fluoroscopy is acquired at the end-diastolic phase per single beat in order for the software to match the baseline reference angiogram with the IVUS transducer location. After image acquisition, based on the distance between the IVUS and the already-inserted guiding catheter in the vessel of reference, an estimate of the association of the fluoroscopic-IVUS image is created via the software, resulting in a co-registered image [43]. The key difference in ULPCI is to use an intracoronary wire angiographic view instead of an angiographic image, which will allow a zero-contrast intervention. This technique allows the association of IVUS-recognized lesions with their respective locations in the baseline angiogram while limiting the injection of contrast.

## 4. Intravascular Imaging for ULPCI—Clinical Data

The use of intravascular imaging, and specifically IVUS, in low-contrast settings has been explored in a number of clinical studies, which are reported in Table 1.

The largest clinical trial to date is MOZART [36]. In particular, Mariani et al. studied the feasibility and safety of an IVUS-guided ULPCI, compared to an angiographic procedure, in a randomized fashion, enrolling 83 patients who underwent either an angiography-guided PCI (n = 42) or an IVUS-guided strategy (n = 41). The study also included more complex lesions, as most of them were categorized as American College of Cardiology (ACC)/American Heart Association (AHA) Type B2 or C. The investigators reported that the use of IVUS resulted in significantly lower total contrast volume, volume of contrast per stent implanted, and contrast volume/creatinine ratio (*p* < 0.001 for all). Furthermore, the study did not report any significant difference in in-hospital or 4-month follow-up patient outcomes, while the only parameter where an IVUS strategy was inferior to angiography was procedure time (*p* = 0.006). Thus, this trial provided encouraging results for the use of IVUS in ULPCI settings; however, the small study cohort warranted more research in order to further establish the potential benefit of IVUS in ULPCI.

Sacha et al. [44], in their respective study, examined the assistance of IVUS in zero-contrast interventions. The study included 20 patients with severe renal disease and under hemodialysis, and a total of 29 PCIs were performed under the guidance of IVUS, aiming for zero-contrast administration. The study showed that the average contrast volume injected was near zero [5 (3.5–9)], while the percentage of AKI following the procedure was 10%, which was lower than the median prediction of 26%, and none of the patients required renal replacement therapy. Moreover, 3 out of 4 hemodialysis patients retained their residual renal function. The study also showed that there were no procedure-related adverse patient outcomes, both post-procedurally and at the time of the follow-up (median 3.2 months).

Further studies also examined the implementation of IVUS in ULPCI strategies; however, they did not examine IVUS as the sole intervention for such strategies. Azzalini et al. [39] studied 111 patients, out of whom 8 underwent ULPCI (4 IVUS, 4 dextran-based OCT, 1 IVUS, and OCT) versus 103 patients undergoing routine angiography-based PCI. There were no differences between the groups regarding lesion complexity, and most lesions were complex (ACC/AHA Type B2 or C). Technical success was achieved in all patients; however, ULPCI was not feasible in 1 out of the 8 patients (IVUS group) due to a procedural complication involving the IVUS catheter. The contrast volume in the ULPCI cohort was significantly lower than the angiography cohort (*p* < 0.001), and the incidence of CIN was lower for ULPCI patients (0% vs. 15.5%), but this difference did not reach statistical significance (*p* = 0.28). Thus, this study shows that intravascular imaging, including IVUS and dextran-based OCT, is a safe and feasible method, in order to decrease the incidence of CIN.

CONSAVE-AKI was a randomized trial aiming to examine the feasibility of ULPCI procedures in AKI patients presenting with acute coronary syndrome (ACS) [45]. The study showed that a ULPCI, in general, is superior to angiography, as the primary outcome of contrast-induced AKI was found to be significantly higher in the angiography cohort (0 vs. 7 patients, *p* = 0.012). However, it is worth noting that not all patients underwent IVUS as their ULPCI strategy. In particular, only 17% of the ULCPI patients underwent the procedure with the assistance of IVUS. Regarding this subgroup, the study showed a reduced contrast volume (29 mL) compared to both the ULPCI full study cohort (41 mL) and angiography (112.5 mL), while the mean contrast volume/eGFR ratio was found to be 1.00. Therefore, this study also adds to the evidence regarding the safety and feasibility of ULPCI in ACS patients, specifically IVUS, which was associated with decreased contrast volume compared to the full ULPCI cohort and could be further associated with the superiority of intravascular imaging for these procedures in comparison to other contrast-limiting techniques.

Ali et al. [42], in their study, examined the effect of using a ULPCI physiology- and imaging-guided strategy in patients with advanced renal failure. In particular, the operators used IVUS as guidance for the coronary intervention and fractional flow reserve measurement to confirm the physiological improvement of the lesion. A total of 31 patients were ultimately included in this study, which showed decreased values of injected contrast media during the baseline angiogram and no-contrast use during the staged PCI procedure. The follow-up examination (median 79 days) also showed no increase in creatinine or eGFR levels, while no patient required renal replacement therapy. Therefore, this study showed that a no-contrast staged PCI procedure, with the use of IVUS and physiology, is feasible and results in enhanced patient outcomes in a high-risk CIN cohort. 

More trials assessed the use of IVUS in no-contrast ULPCI settings. Kumar et al. [37] assessed 42 CKD patients most commonly presenting with ACS who underwent zero-contrast PCI in order to determine the safety and short-term outcomes of the procedure. Out of the 66 vessels treated, 14 (21.2%) were left main coronary arteries, and 3 (4.5%) had chronic total occlusions, aiming to examine the safety of the technique in more complex lesions. Technical success was achieved in 61 vessels (92.4%), as 5 vessels were treated under ULPCI parameters. Peak creatinine at 48 h post-operatively was not different from the pre-operative value. Regarding outcomes, 1 patient suffered from a procedure-related event and 4 required blood transfusion, while regarding renal outcomes, 5 patients developed CIN, per the KDIGO definition; however, none required dialysis or any further treatment.

Followingly, Shibata et al. [38], in their study, examined the long-term safety and efficacy of IVUS-guided zero-contrast PCI in CKD patients. In particular, they assessed 517 patients, out of whom 55 underwent zero-contrast PCI and 462 underwent conventional PCI, which were then propensity matched in 50 patients per cohort. There was no difference regarding the complexity of the lesions and left main revascularization between the two arms. The study showed that an IVUS-guided approach was successful in all patients, while no renal complications occurred in any of the two groups. Moreover, at the mean follow-up for each study arm, there were no differences observed in regards to major adverse cardiovascular outcomes, all-cause cardiovascular death, or renal replacement therapy, thus confirming the safety and efficacy of IVUS zero-contrast PCI, compared to angiography, in a longer-term follow-up.

Finally, Nandhakumar et al. [46], in a prospective study, enrolled 27 patients at risk for CIN (31 vessels) and assessed the technical success of IVUS-guided zero-contrast PCI and adverse outcomes at 30 days follow-up. Technical success was met in 87.1% (n = 27) of treated vessels. However, no major adverse cardiac or cerebrovascular event was documented at 30 days follow-up, and the initiation of renal replacement therapy was not required until the end of the follow-up period. The authors conclude that, despite the fact that the technique is safe and efficient in selective coronary anatomies, small injections of contrast may be necessary when performing such interventions in order to overcome complications such as slow flow, intraprocedural thrombus, and geographical miss.

## 5. Future Perspectives

**Figure 4:**
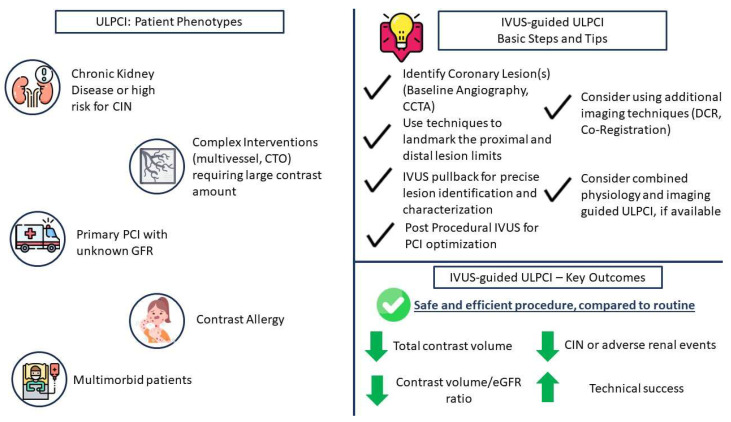
Central figure: Ultra-low percutaneous coronary intervention (ULCPI) and intravascular ultrasound (IVUS): patient characteristics, technical considerations and key outcomes. Abbreviations: CIN: contrast-induced nephropathy, CTO: chronic total occlusion, PCI: percutaneous coronary intervention, CCTA: coronary computed tomography angiography, DCR: dynamic coronary roadmapping, eGFR: estimated glomerular filtration rate.

Intravascular imaging, and particularly IVUS, plays a key role in ULPCI intervention. As documented from the aforementioned clinical trials, the use of IVUS to guide coronary interventions led to a significant reduction in contrast volume, contrary to conventional angiography, while the effectiveness and safety of the procedure were comparable to angiography, as increased mortality and adverse outcome rates were not observed. Furthermore, the use of IVUS was shown to be safe in the context of zero-contrast PCI, with no expense to the technical success of the procedure. These results imply that such techniques, employed to avoid unnecessary contrast use, can be safely used in patients at high risk for CIN or any other severe contrast-mediated adverse reaction (Figure 4: central figure). Especially for patients with CKD or pre-intervention renal failure of other etiologies, such as shock, the use of imaging-only interventions seems to be ideal for avoiding contrast-associated complications. Furthermore, for patient phenotypes where the contrast volume is expected to be large (i.e., CTO and complex intervention), regardless of CIN risk, ULPCI creates the opportunity for both the patient and operator to limit the use of contrast and perform revascularization in one attempt rather than stage-up the operation, while, as shown in the aforementioned studies, IVUS-guided ULPCI can be performed safely in such lesions. Finally, limited data also show the safety and efficacy of ULPCI even in ACS patients, who, as shown by the Mehran 2 risk score, are also at high risk for CIN. It should be highlighted, though, that the data supporting the use of ULPCI or zero-contrast PCI remain limited to mostly observational studies, with no extended follow-up. Furthermore, only a few trials have examined the sole use of IVUS in these procedures, while most trials have tested the combined use of ULPCI strategies, including physiology assessment and contrast dilution. Indeed, in everyday clinical practice, a combination of ULPCI techniques is more probable to be used by operators in order to ensure a safe procedure. However, in order for such interventions to be more widely used and ultimately endorsed by guidelines, more randomized trials with extensive follow-up, examining the specifics of IVUS use in this setting are needed. Currently enrolling randomized trials in patients with CKD and AKI (NCT05913362, NCT05906758, and NCT02743156) will provide more insights regarding the use of IVUS-guided ULPCI in these patients.

Novel technologies in imaging-guided ULPCI warrant the expansion of the field and the employment of new techniques, which have the potential to improve patient outcomes and further assist in optimizing coronary interventions. In particular, as aforementioned, OCT is currently not the preferred imaging modality for guiding ULPCI, as the need for contrast in order to acquire intravascular images is a prohibiting factor for this type of intervention. However, it is also known that, despite the advances in IVUS technology and the technical enhancement of the acquired images over the years, OCT has better discrimination capabilities as well as resolution, resulting in better intravascular image acquisition [40]. The use of zero- or ultra-low contrast PCI under OCT guidance, employing agents such as dextran and colloid infusate, has been described in the bibliography, with the reported outcomes being favorable for the method [47,48,49]. However, the lack of concrete clinical evidence employing this method warrants further research and clinical investigations in order to first evaluate the safety and feasibility of the method and, second, to compare OCT-guided ULPCI to IVUS. 

Finally, co-registration has been proven increasingly beneficial for performing ULPCI. It should be, thus, mentioned that the operators’ option for co-registering the angiographic image includes both physiology co-registration, where the fluoroscopy image is co-registered with physiology-derived data, and imaging co-registration, where the fluoroscopy image is co-registered with IVUS pullback data. However, specific software used for co-registration can also allow tri-registration, i.e., co-registration of fluoroscopy, physiology, and imaging data. The use of such software allows a total evaluation of the coronary arteries and potential lesions and provides the most data to physicians, even in the absence of the typical angiographic image. Despite the lack of clinical data regarding tri-registration, future trials should focus on the evaluation of such systems in order for them to be more widely implemented in clinical practice.

## 6. Conclusions 

Intravascular imaging is a significant contributor to the success of ULCPI procedures. The available clinical data, even though limited, document both the efficiency and safety of a ULPCI under the guidance of IVUS. Furthermore, the emergence of newer OCT-based algorithms using non-iodinated contrast could also be associated with enhanced patient outcomes and coronary artery visualization. Thus, future, larger randomized trials testing both IVUS and OCT in this setting could increase the evidence and the clinical use of such procedures in selected patients, resulting in improved PCI procedures.

## Figures and Tables

**Table 1:** Clinical trials involving the use of IVUS in ULPCI or zero-contrast strategies.

Clinical Trial	Type of Study	Year	Patient Characteristics	Technique	Outcomes
Mariani et al. [36]	Randomized controlled trial	2014	83 CKD patients undergoing either angiography-guided PCI (n = 41) or ULPCI	ULCPI, IVUS guided	The total contrast volume was significantly reduced in IVUS-guided ULPCI (64.5 mL vs. 20.0 mL, *p* < 0.001) Contrast volume/creatinine ratio was significantly lower in ULPCI (1.0 vs. 0.4, *p* < 0.001) No significant difference in the creatinine levels post-operatively or the incidence of CIN
Sacha et al. [44]	Observational	2019	20 patients with advanced CKD and hemodialysis (29 lesions) undergoing ULPCI	Zero-contrast IVUS guided	The technique was feasible in all lesions Post-operative AKI rate was 10% No patient required renal replacement therapy (RRT)No patient experienced coronary-related adverse events at 3 months median follow-up
Azallini et al. [39]	Observational	2019	111 patients with CKD (ULPCI: n = 8, conventional angiography: n = 103)	ULPCI, IVUS and/or OCT guided	Contrast volume was significantly lower in the ULPCI group (8.8 mL vs. 90 mL, *p* < 0.001) Technical success was 100% ULPCI protocol success was 88% The incidence of CIN was lower in the ULPCI, but not statistically significant (0% vs. 15.5%, *p* = 0.28)
Shrivastava et al. (CONSAVE-AKI) [45]	Randomized controlled trial	2022	82 patients with acute coronary syndrome at high risk for CIN (ULPCI: n = 41, conventional strategy: n = 41) 7 patients underwent IVUS-guided ULPCI	ULPCI, including contrast dilution and IVUS techniques	Contrast volume was lower for IVUS-ULPCI (29 mL), contrary to the total ULPCI cohort (41 mL) and the angiography arm (112.5 mL Contrast volume/eGFR ratio was similar between the ULPCI total cohort (0.97) and the IVUS-guided arm (1.00), while being lower than the conventional strategy (2.68) No CIN was evident in ULPCI patients, in contrast to the typical angiography-guided PCI (17.1%)
Ali et al. [42]	Observational	2016	31 patients with advanced CKD, who had PCI indication in a prior coronary angiogram	Zero-contrast, IVUS and physiology guided	Contrast volume was consistently low in all patients (median 13 mL), as was the contrast volume/eGFR ratio (0.80) No significant changes were observed in post-operative creatinine and eGFR levels No patient experienced an adverse outcome related to the procedure or needed RRT
Kumar et al. [37]	Observational	2022	42 CKD patients (66 vessels) with an indication for absolute zero-contrast PCI	Zero-contrast, IVUS guided	Technical success of zero contrast PCI was 92.4% (61 lesions) 5 lesions (7.6%) were revascularized under ULPCI conditions 5 patients (11.9%) developed AKI; however, none required RRT or other treatment No procedure-related mortality was observed in both short and long (1 year) follow-up
Shibata et al. [38]	Observational	2022	100 propensity matched CKD patients undergoing PCI (50 patients in each of the following strategies: IVUS-guided zero-contrast PCI, conventional angiography)	Zero-contrast, IVUS guided	Zero-contrast PCI was successful in all patients No AKI events were documented in both cohorts post-operatively There was no statistical difference between the major adverse events or mortality between the two cohorts
Nandhakumar et al. [46]	Observational	2022	27 patients with CKD (31 vessels)	Zero-contrast, IVUS guided	The technical success of the zero-contrast procedure was 87.1% (27 lesions) At one month follow-up, no patient required RRTT here were no recorder major adverse cardiovascular or cerebrovascular events

## Data Availability

No original data were used in this literature review.
